# Mesoporous Silica Nanoparticles as a Potential Platform for Vaccine Development against Tuberculosis

**DOI:** 10.3390/pharmaceutics12121218

**Published:** 2020-12-16

**Authors:** Sandra Montalvo-Quirós, María Vallet-Regí, Ainhoa Palacios, Juan Anguita, Rafael C. Prados-Rosales, Blanca González, Jose L. Luque-Garcia

**Affiliations:** 1 Department of Analytical Chemistry, Faculty of Chemistry, Complutense University of Madrid, 28040 Madrid, Spain; smquiros@ucm.es; 2 Centro de Estudios Tecnológicos y Sociales y Facultad de Experimentales, Francisco de Vitoria University, 28223 Madrid, Spain; 3 Department of Chemistry in Pharmaceutical Sciences, Faculty of Pharmacy, Instituto de Investigación Sanitaria Hospital 12 de Octubre (imas12), Complutense University of Madrid, 28040 Madrid, Spain; vallet@ucm.es; 4 Centro de Investigación Biomédica en Red de Bioingeniería, Biomateriales y Nanomedicina (CIBER-BBN), 28029 Madrid, Spain; 5 Inflammation and Macrophage Plasticity Lab, CIC bioGUNE, 48160 Derio, Spain; apalacios@cicbiogune.es (A.P.); janguita@cicbiogune.es (J.A.); 6 Ikerbasque, Basque Foundation for Science, 48009 Bilbao, Spain; 7 Department of Preventive Medicine and Public Health and Microbiology, Faculty of Medicine, Autonomous University of Madrid, 28049 Madrid, Spain; rafael.prados@uam.es

**Keywords:** *Mycobacterium tuberculosis*, mesoporous silica nanoparticles, immunomodulatory proteins, immune system activation, tuberculosis vaccines

## Abstract

The increasing emergence of new strains of *Mycobacterium tuberculosis* (*Mtb*) highly resistant to antibiotics constitute a public health issue, since tuberculosis still constitutes the primary cause of death in the world due to bacterial infection. *Mtb* has been shown to produce membrane-derived extracellular vesicles (EVs) containing proteins responsible for modulating the pathological immune response after infection. These natural vesicles were considered a promising alternative to the development of novel vaccines. However, their use was compromised by the observed lack of reproducibility between preparations. In this work, with the aim of developing nanosystems mimicking the extracellular vesicles produced by *Mtb,* mesoporous silica nanoparticles (MSNs) have been used as nanocarriers of immunomodulatory and vesicle-associated proteins (Ag85B, LprG and LprA). These novel nanosystems have been designed and extensively characterized, demonstrating the effectiveness of the covalent anchorage of the immunomodulatory proteins to the surface of the MSNs. The immunostimulatory capacity of the designed nanosystems has been demonstrated by measuring the levels of pro- (TNF) and anti-inflammatory (IL-10) cytokines in exposed macrophages. These results open a new possibility for the development of more complex nanosystems, including additional vesicle components or even antitubercular drugs, thus allowing for the combination of immunomodulatory and bactericidal effects against *Mtb*.

## 1. Introduction

*Mycobacterium tuberculosis* (*Mtb*) remains a global health issue accounting for significant morbidity and mortality worldwide. In 2019, an estimated 2 million deaths occurred from tuberculosis (TB) [1]. *Mtb* is so infrequently eliminated by the host that an estimated one-third of humanity is latently infected for life. Among those initially infected, a small percentage (5% to 10%) fail to control the initial infection and develop primary TB disease. The majority of humans infected with this microorganism mount an effective immune response that results in latent infection. However, reactivation of latent infection can occur years to decades later in a small subset of infected persons, leading to active TB. Despite the availability of a treatment and a vaccine, TB is still the world’s leading cause of death from a bacterial infection and paradoxically from a curable infection [1]. Efforts to control the disease include the development of point-of-care tests, new TB drugs, the use of the BCG vaccine, and the development of new vaccines [2]. The capacity of *Mtb* to persist in hosts with intact immunity and avoid immune-mediated clearance reflects an evolved and coordinated program of immune evasion strategies. The understanding of the mechanisms that *Mtb* uses to escape from effective immune responses is of paramount importance to develop effective treatments. *Mtb* was shown to produce membrane-derived extracellular vesicles (EVs) that are highly immunogenic and elicit immune responses dependent of Toll-like receptor 2 [3]. *Mtb* EVs have a complex composition including glycolipids and many proteins, some which have been identified as immunodominant antigens, such as lipoproteins and Ag85B [3]. Although the mechanism by which such vesicles are produced and exported across the mycobacterial cell wall is not known, similar vesicles have been described in other microbes with dense outer membranes walls, such as Gram-positive bacteria and fungi [4]. When *Mtb* EVs are instilled in the lung of mice followed by aerosolized infection they elicit a “Koch-phenomenon” with enhanced inflammatory damage [3]. However, when these vesicles are given intraperitoneally or subcutaneously before infection, they elicit protective responses that are comparable to those elicited by BCG vaccination [5]. The fact that EVs, a mycobacterial component, elicit immune responses that are comparable to BCG with regards to protection is exciting, because it means that it may be possible to design vesicle-derived vaccines that lack the problems associated with the use of live vaccines. Nevertheless, there are still some practical aspects of their production that need to be tailored, since variable protection of independent EVs batches produced under the same conditions was observed [5]. These observations suggest that naturally produced EVs might not represent an ideal vaccine candidate. On the other hand, the direct administration of the immunomodulatory proteins has the problem of the low physico-chemical stability of proteins under physiological conditions until they reach their target. In addition, intracellular delivery of active proteins into specific cells may be desired and several proteins are usually unable to cross cell membranes. To solve both problems, artificial *Mtb* EVs incorporating recombinantly expressed MEV-associated proteins could be developed and tested as an alternative vaccine [6].

Nanotechnology is a science in growing development with increasingly relevant applications in biomedicine [7,8,9,10]. Several authors have already designed novel nanosystems to fight against TB, both with bactericidal purposes against *Mtb* [11,12,13] and nanosystems oriented to the development of new vaccines [14]. In addition, some nanotechnological strategies for functional protein delivery have been recently developed achieving protein protection and an improved therapeutic effect [15]. Some of the approaches deal with protein conjugation with polymers, protein entrapment into the aqueous core of lipidic vesicles, wrapping of proteins into polymeric nanocapsules, and the transport of proteins attached onto the surface of inorganic nanocarriers or inside their structure. In this sense, mesoporous silica nanoparticles (MSNs) are inorganic nanomaterials widely recognized as versatile drug delivery systems due to their unique properties [16,17,18,19]. These nanocarriers possess tunable particle size in the 50–200 nm range and narrow pore size distributions of 3–6 nm, large surface areas and pore volumes, and remarkably high biocompatibility [20]. Furthermore, MSNs have also been investigated as protein carriers either transporting the proteins trapped in their porous structures or located on the external surface, in the last case directly attached or even via the coupling of protein protecting polymeric nanocapsules on the MSNs surface [21,22,23,24]. While MSNs have already been used mainly as nanocarriers of anti-tuberculosis drugs [25,26,27], they also have been investigated in vaccine development as antigen carriers [28] as well as acting as adjuvants [29]. Furthermore, in vivo studies have shown MSN safety, immunogenicity, and protection against some pathogens [30,31,32,33,34]. Therefore, MSNs represent a very attractive platform for the transport of immunodominant antigens that mimic the *Mtb*’s naturally produced EVs.

Based on all of the above, this work focuses on the development of novel nanosystems based on the use of MSNs as nanocarriers of immunomodulatory and vesicle-associated proteins (Ag85B, LprG and LprA) against TB. After the synthesis of the nanosystems and a deep analytical characterization, their cytotoxicity and immunostimulatory capacity is investigated.

## 2. Materials and Methods

### 2.1. Reagents and Equipment

An inert atmosphere was used for the reactions of chemical modification of the silica surface. Fluorescein isothiocyanate (FITC), tetraethyl orthosilicate (TEOS), cetyltrimethylammonium bromide (CTAB), *N*-(3-Dimethylaminopropyl)-*N*′-ethylcarbodiimide hydrochloride (EDC.HCl), 2-morpholinoethanesulfonic acid (MES), Bradford reagent, lipopolysaccharides from *Escherichia coli* (LPS) and water (HPLC grade) were purchased for Sigma-Aldrich (Madrid, Spain). 3-Aminopropyltriethoxysilane (APTS) and 3-(triethoxysilyl)propylsuccinic anhydride (TESPSA) were purchased from ABCR GmbH (Karlsruhe, Germany) and Co.KG. *M. tuberculosis* Ag85B, LprG and LprA proteins were recombinantly expressed in *E. coli* as previously reported [35]. All recombinant proteins were extensively purified by size-exclusion chromatography to achieve non-detectable levels of LPS. The amount of residual LPS in the protein preparations was evaluated using a Limulus Amebocyte Lysate (LAL) test kit (Lonza, Basel, Switzerland) according to the manufacturer’s instructions. Purified endotoxin-free *M. tuberculosis* proteins were filter sterilized and frozen at −70 °C.

The analytical methods used to characterize the synthesized compounds were as follows: Mass spectrometry (MS), thermogravimetric and differential thermal analysis (TGA), chemical microanalyses, solid state magic angle spinning (MAS) NMR and cross polarization (CP) MAS NMR spectroscopy, low-angle powder X-ray diffraction (XRD), N_2_ adsorption porosimetry, electrophoretic mobility measurements to calculate the values of zeta-potential (ζ), dynamic light scattering (DLS), scanning electron microscopy (SEM), and transmission electron microscopy (TEM). The equipment and conditions used are described in the Supporting Information.

### 2.2. Materials Synthesis

The external surface functionalization of MSNs with carboxylic acids was performed onto the pore surfactant containing material (34.6% wt.). Approximately a quarter of the specific surface area of the free-surfactant material (1368.4 m^2^/g) was considered to be functionalized [36]. First, 1 g of CTAB-containing MSNs (0.654 g MSNs) was dehydrated at 80 °C under vacuum for 3 h in the dark. Subsequently, TESPSA (0.2032 g, 10% exc.) was dissolved in 15 mL of dry toluene and added to a vigorously stirred suspension of the CTAB-containing MSNs dispersed in dry toluene (60 mL), under N_2_ atmosphere. The reaction mixture was heated to 110 °C overnight in the dark. Afterwards it was centrifuged and the modified MSNs were exhaustively washed with toluene and acetone and finally dried. The surfactant extraction was carried out by heating a well-dispersed suspension of the obtained solid in EtOH (360 mL), water (40 mL) and HCl (10 mL) overnight at 60 °C. Finally, the solid was washed with water and EtOH. This process was repeated for 2 h and the solid dried under vacuum. The experimental value of –COOH groups in this material is 6.74 × 10^−4^ mol/g MSN-COOH_ext_, calculated from the organic content (5.4%) due to the functionalization, as determined by thermogravimetry.

Prior to the conjugation of the immunomodulatory proteins, the carboxylic acid groups on the surface of MSNs-COOH_ext_ were activated. For this activation, different amounts of MSNs-COOH_ext_ (0.1122 g, 0.2615 g and 0.1705 g, respectively for each Ag85B, LprG and LprA protein anchorage) were well dispersed in water and, subsequently, EDC.HCl (10 equiv per –COOH group) was dissolved in water and added over the MSNs-COOH_ext_ suspension. The mixture was stirred at room temperature for 3 h in the dark. Then, the solid was centrifuged and rinsed with water to remove the residuals of the activating agent. The amount of MSNs-COOH_ext_ material was calculated taking into account the mass of the protein and the following molar ratio 1.4 × 10^−3^ mol protein per –COOH mol, as previously established in our former work [22]. Activated MSNs-COOH_ext_ were re-dispersed in MES monohydrate (50 mM, pH depending on the protein) under gently stirring. After that, each protein (3.4 mg of Ag85B (1.0586 × 10^−7^ mol), 5.6 mg of LprG (2.4171 × 10^−7^ mol), and 3.9 mg of LprA (1.6238 × 10^−7^ mol)) was dissolved in MES monohydrate 50 mM (pH 4.8, pH 6.7, and pH 4, respectively, for each protein) and added over the material suspension. The mixture was stirred overnight in the dark, centrifuged, and the solid was washed and finally dried. The nanosystems were denoted as MSNs-Ag85B, MSNs-LprG, and MSNs-LprA, respectively.

### 2.3. Cytotoxicity Assay

RAW 264.7 cells were seeded on 96-well plates and exposed to 5, 10, and 50 μg/mL of the nanomaterials for 24 and 48 h. After each contact time, 20 μL of 3-(4,5-dimethyl-thiazol-2-yl) 2,5-diphenyl tetrazolium bromide (MTT, 5 mg/mL) were added to each well and incubated for 5 h at 37 °C. Then, the MTT solution was removed and 100 μL of dimethyl sulfoxide were added to dissolve the insoluble purple formazan products. The absorbance at a 595 nm was determined using a microplate reader (TECAN) and the cell viability calculated through the relation between the absorbance of treated cells and the absorbance of control cells. The final results were calculated on the basis of 5 replicates of the experiment.

### 2.4. ELISA

RAW 264.7 cells were seeded in P60 plates and stimulated with 10 μg/mL of MSNs-Ag85B, MSNs-LprG or MSNs-LprA for 24 h. Lipopolysaccharide (LPS) from *Escherichia coli* was used as positive control. Cells were exposed to a solution of LPS at 100 ng/mL for 24 h. After stimulation, cell culture media were collected and frozen. To evaluate the levels of the pro-inflammatory cytokine TNF, and the anti-inflammatory cytokine, IL-10, ELISA procedures were used according to the manufacturer’s instructions (mouse TNF DuoSet ELISA kit, RyD systems; mouse IL-10 ELISA kit, Novex Life Technologies (Madrid, Spain). This experiment was carried out in triplicate.

## 3. Results and Discussion

### 3.1. Immunomodulatory Proteins

With the aim of mimicking naturally produced EVs, we selected Ag85B, LprG and LprA proteins based on the facts that: (i) They were identified in *Mtb*’s EVs [3,37], (ii) they were shown to be responsible for the immunogenicity of similar EVs [5]; and they are common immunodominant antigens in *Mtb*. Such proteins were characterized by MALDI-TOF mass spectrometry prior to their anchorage on the external surface of the mesoporous silica nanoparticles. The mass spectrum of the Ag85B protein exhibited a peak at m/z 32,117.49, which corresponded to the molecular peak of the Ag85B antigen (Figure S1A). In the case of the mass spectra obtained for the two lipoproteins, LprG (Figure S1B) and LprA (Figure S1C), both the molecular peak at m/z values of 23,168.44 and 24,017.55, respectively, as well as peaks at m/z values corresponding to doubly and triply charged peaks with respect to molecular mass, were observed. This characterization allowed confirming the expected molecular weight of the proteins obtained which presented a high level of purity.

### 3.2. Synthesis and Characterization of the Immunomodulatory Nanosystems

**Figure 1:**
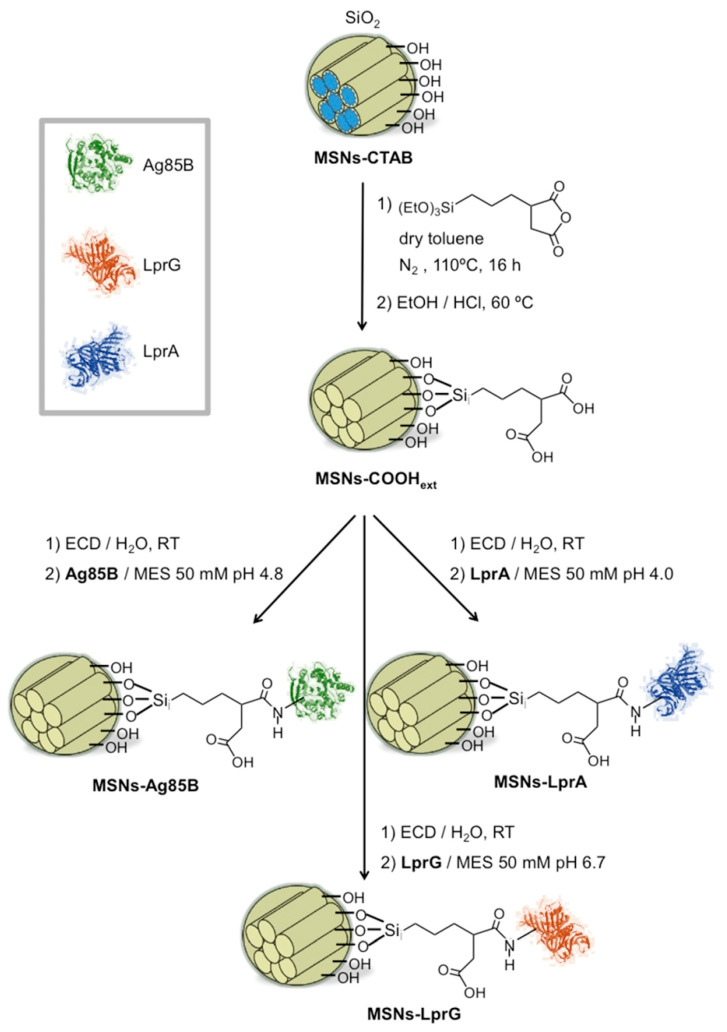
Scheme of synthesis of mesoporous silica nanoparticle (MSN) materials functionalized with the immunomodulatory proteins Ag85B, LprG, LprA.

Figure 1 shows the synthetic route to obtain three hybrid nanosystems consisting of MSNs externally functionalized with proteins from *Mtb* (MSNs-Prot*_Mtb_*). MSNs (Figure S2A–D) were first functionalized with carboxylic acids on the external surface following a post-synthetic approach under water free conditions and using the surfactant containing MSNs to favor the distribution of the alkoxysilane on the outer MSNs surface [38,39]. In a second step, the proteins were covalently grafted to the MSNs-COOH_ext_ material through stable amide bonds following our previous methodology [22]. The carboxylic acid groups were activated and condensed through carbodiimide chemistry with some of the free primary amino groups on the *Mtb* proteins, present in lysine and arginine amino acids. Considering the steric hindrance effects that take place when macromolecules are employed to functionalize the silica surface, the amount of MSNs-COOH_ext_ material was calculated taking into account the mass of the protein and the molar ratio 1.4 × 10^−3^ mol protein per –COOH mol. This methodology allowed us to use in the synthesis the amount of protein that is closest to the amount that can be anchored due to steric factors. Another important factor to consider for anchoring the proteins onto the surface of the silica is the isoelectric points of the proteins and the pH of the reaction. Therefore, the pH of the condensation reaction was adjusted below the isoelectric point of the proteins (IEP_Ag85B_ 5.62 [40], IEP_LprG_ 7.78 [41], and IEP_LprA_ 5.00 [42]), leading to the protonation of their amino acids residues and avoiding electrostatic repulsion with the negative surface of the silica nanoparticles (MSNs-COOH_ext_) [43].

Thermogravimetric analysis was performed to confirm the incorporation of the carboxylic acids and the proteins in the sequential synthetic steps (Table 1). An expected increase of the organic content with respect to bare MSNs was produced for the MSNs-COOH_ext_ due to the alkoxysilane incorporation. Additionally, for the three MSN-Prot*_Mtb_* materials, there was an increase of the organic content with respect to the carboxylic acid functionalized MSNs due to the proteins, which was very similar for the three materials. Furthermore, the quantification of the non-attached protein was measured by the Bradford assay to determine the efficiency of anchorage. The absorbance due to the free protein in the supernatant of the reaction medium was used to compare the concentration of the remaining free protein after the attachment with the concentration of the protein added in the reaction. As shown in Table 1, the efficiency of anchoring was 99% for MSNs-Ag85B and MSNs-LprG and 95% for MSNs-LprA.

The zeta-potential (ζ) values of the nanosystems in water media were measured to follow the functionalization process (Table 2 and Figure S3). Bare MSNs showed a negative ζ-potential due to the presence of –SiO^−^ groups of silica surface according to Equation (1). A shift to more negative ζ-potential value compared to the bare MSNs was produced after the grafting of TESPSA alkoxysilane, ascribed to the presence of negative –COO^−^ groups from the carboxylic acid functionalities (Equation (2)) [44]. The subsequence introduction of proteins onto MSNs-COOH_ext_ did not affect much, provoking a slightly change towards less negative ζ-potential. These values reflect that only few –COOH groups reacted to form amide bonds because of steric hindrance effects of the proteins. Furthermore, the anchored proteins must be above or close to their isoelectric point in the water media, hence being negatively charged or close to neutral and; therefore, not influencing the final value much.

R-Si-OH + H_2_O ⇌ R-SiO^−^ + H_3_O^+^ pK_a_ ≈ 6.8 (1)

R-COOH + H_2_O ⇌ R-COO^−^ + H_3_O^+^ pK_a_ ≈ 4.8 (2)

The hydrodynamic size of the nanosystems was assessed through dynamic light scattering (DLS) measurements of the materials in water medium (Table 2 and Figure S3). All materials showed monomodal hydrodynamic size distributions centered around 200 nm, in accordance with the negative ζ-potential values, which are high enough for the nanoparticles to be in the colloidal stability zone [45]. The maximum of the hydrodynamic size distribution was not significantly altered from the bare MSNs to the carboxylic acid and the *Mtb* proteins functionalized nanosystems, although alkoxysilane grafting slightly increased the hydrodynamic size of the MSNs. Nevertheless, when the *Mtb* proteins were attached to the external surface of MSNs-COOH_ext_, a shift to smaller values was produced, reflecting the contribution of steric effects due to the presence of organic macromolecules to the electrostatic repulsion and therefore decreasing the size of the aggregates in solution.

**Figure 2:**
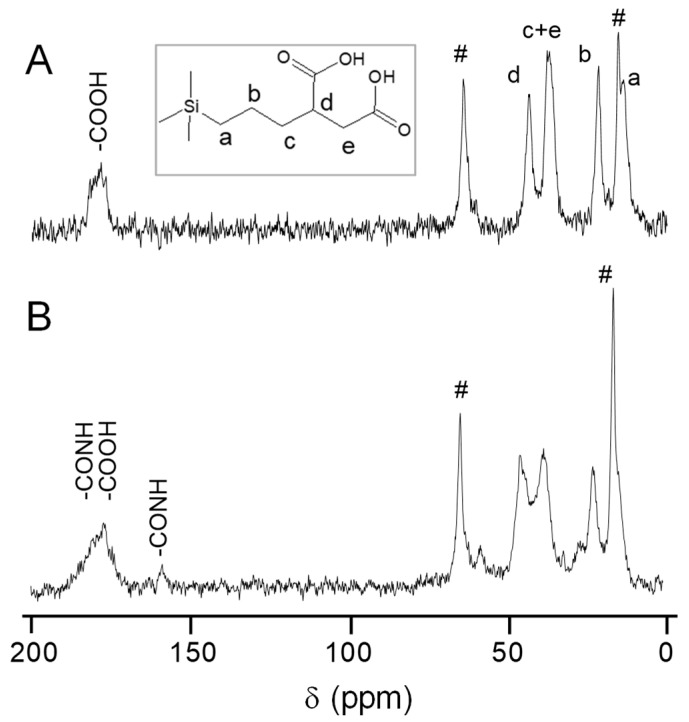
^13^C {^1^H} cross polarization (CP) MAS NMR spectra of materials: (**A**) MSNs-COOH_ext_ and (**B**) MSNs-LprG. Peaks designated with # correspond to ethoxy groups due to incomplete hydrolysis and condensation.

Solid-state MAS NMR spectroscopy was used to further analyze the functionalization of mesoporous silica nanoparticles. Spectra of MSNs, MSNs-COOH_ext_ and MSNs-Prot*_Mtb_* materials in the sequence of synthesis were recorded, using MSNs-LprG as a representative example of MSNs-Prot*_Mtb_*. All the ^13^C {^1^H} CP MAS NMR spectra of the nanosystems (Figure 2 and Figure S4) exhibited two peaks at 64 and 15 ppm, corresponding to methylene and methyl groups, respectively, ascribed to the ethoxy groups from incomplete hydrolysis and condensation of the tetraethyl orthosilicate precursor during the sol-gel synthesis of MSNs [46]. The MSNs-COOH_ext_ material spectrum (Figure 2a) showed a broad signal at ca. 178 ppm corresponding to the carbonyl moiety of the carboxylic acid groups. Then, the CH adjacent to the carboxylic acid group showed a peak at 43 ppm (signal d) and the peak at 37 ppm corresponding to the sum of CH_2_ groups closed to the carboxylic acid group (signal c and e). The peak at 21 ppm matched with the methylene carbon in the chain (signal b) and, finally, the methylene carbon directly attached to silicon atom showed a peak at 13 ppm (signal a). Figure 2B shows the spectrum of the MSNs-LprG nanosystem. The carbonyl signal at around 178 ppm broadens because in addition to the remaining carboxylic acid groups from the former MSNs-COOH_ext_, the protein structure contributes with carboxylic acid groups and amide bonds. In addition, a new signal appeared at ca. 155 ppm, which confirms the formation of amide-like bonds corroborating the covalent union between the protein and the nanoparticles.

**Figure 3:**
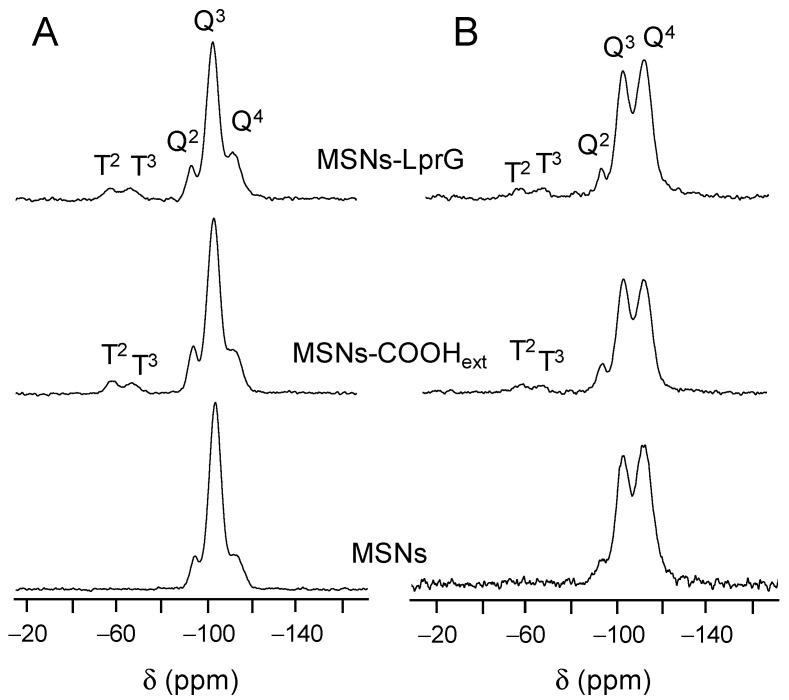
^29^Si cross polarization (CP) MAS NMR spectra (**A**) and ^29^Si MAS NMR spectra (**B**) of MSNs, MSNs-COOH_ext_ and MSNs-LprG materials.

Figure 3 shows solid-state ^29^Si MAS NMR spectra of the bare MSNs, the functionalized MSNs-COOH_ext_ and the MSNs-LprG nanomaterials. Cross-polarized (CP) measurements were registered to assess the existence of T units [R-Si(OSi)_n_(OX)_3−n_] (X = H, C) due to the presence of the functionalizing trialkoxysilane in the materials. Signals at −57 and −67 ppm in Figure 3A come from T^2^ [R-Si(OSi)_2_(OX)] and T^3^ [R-Si(OSi)_3_] units, respectively, of the functionalized MSNs-COOH_ext_ and MSNs-LprG nanomaterials. Furthermore, quantitative measurements (Figure 3B and Table 3) were employed to calculate the populations of the silicon Q^n^ and T^n^ environments. The Q^2^ [Si(OSi)_2_(OX)_2_], Q^3^ [Si(OSi)_3_(OX)] and Q^4^ [Si(OSi)_4_] silicon sites give resonances at ca. −93, −102, and −112 ppm, respectively, in all spectra (X = H, C). As shown in Table 3, after the covalent grafting of the alkoxysilane TESPSA on the silica surface, there was a decrease in the Q^2^ and Q^3^ peak areas and an increase in the Q^4^ population. This effect is due to the conversion from Si-OH to fully condensed Si-O-Si species in MSNs-COOH_ext_. Interestingly, Q^2^ and Q^3^ environments were still preserved, as pointed out by the (Q^2^ + Q^3^)/Q^4^ relative ratio of partially to fully condensed silicon sites, confirming that the inner surface of the mesochannels has been preserved from functionalization. As expected, there was no variation in the populations of Q^n^ sites from MSNs-COOH_ext_ to MSNs-LprG material because the protein is attached without involving silicon sites. The relation of Q^n^/T^n^ environments was also maintained, supporting the anchorage of proteins through the carboxylic acid groups.

**Figure 4:**
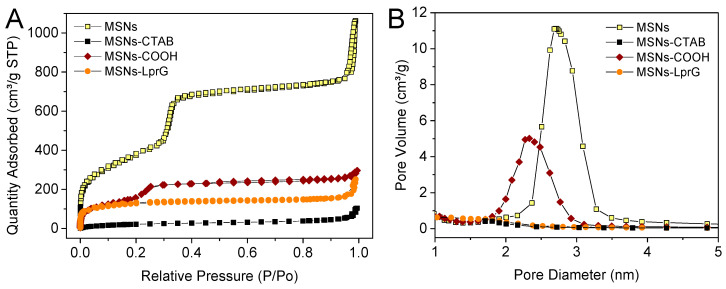
(**A**) N2 adsorption isotherms of the surfactant extracted MSN materials and the cetyltrimethylammonium bromide (CTAB) containing MSNs, before and after functionalization with carboxylic acid groups and protein. (**B**) Pore-size distributions for the mesoporous samples.

The textural properties of these MSNs-based nanosystems were analyzed by N_2_ adsorption-desorption measurements (Figure 4 and Table 4). As expected, the surfactant-containing material (MSNs-CTAB) exhibited a characteristic isotherm of a non-porous material possessing a very low surface area (104.2 m^2^/g). After functionalization with the carboxylic acids and subsequent removal of the surfactant, MSNs-COOH_ext_ showed a higher surface area (557.2 m^2^/g) and a large pore volume (0.274 cm^3^/g). However, these values were lower than values obtained for the bare surfactant extracted MSNs, confirming the alkoxysilane grafting. Pore diameter was 2.73 and 2.03 nm, very similar for both MSNs and MSNs-COOH_ext_ materials, respectively. Furthermore, both materials exhibited type IV Brunauer–Emmett–Teller (BET) isotherms with no observed hysteresis loop, corroborating the presence of a cylindrical one-dimensional channel-like mesoporous structure in the nanoparticles. The N_2_ isotherms present a sharp inflection at a relative pressure of 0.25–0.35 and 0.20–0.30, respectively, for MSNs and MSNs-COOH_ext_ materials, which corresponds to the phenomena of capillary condensation and evaporation within channel-type uniform mesopores. Hence, in concordance with the Si-OH population revealed by ^29^Si MAS NMR analysis, the TESPSA anchorage took place preferable on the external surface instead of the internal surface of the mesopores, since it was performed before the surfactant extraction step. Surface area, pore diameter and pore volume were somehow reduced after the anchorage of the immunomodulatory LprG protein on the external surface of the MSNs-COOH_ext_ (440.6 m^2^/g, 1.20 nm, and 0.218 cm^3^/g). This effect was due to the partial blockage of the entrance of the pores by the macromolecules, which is also in part related to the drying treatment of the material before N_2_ sorption measurement. In addition, a secondary step at a pressure above 0.90 P/P_0_ is observed for all the samples, attributed to condensation in the interparticle porosity, that is, in the macropores formed among the nanoparticles after drying. For that reason, Table 4 includes both V_T_, which is the total pore volume obtained at P/P_0_ = 0.99 and V_P_, which is the total pore volume obtained at P/P_0_ = 0.60 and does not take into account interparticle porosity.

### 3.3. Cell Viability

**Figure 5:**
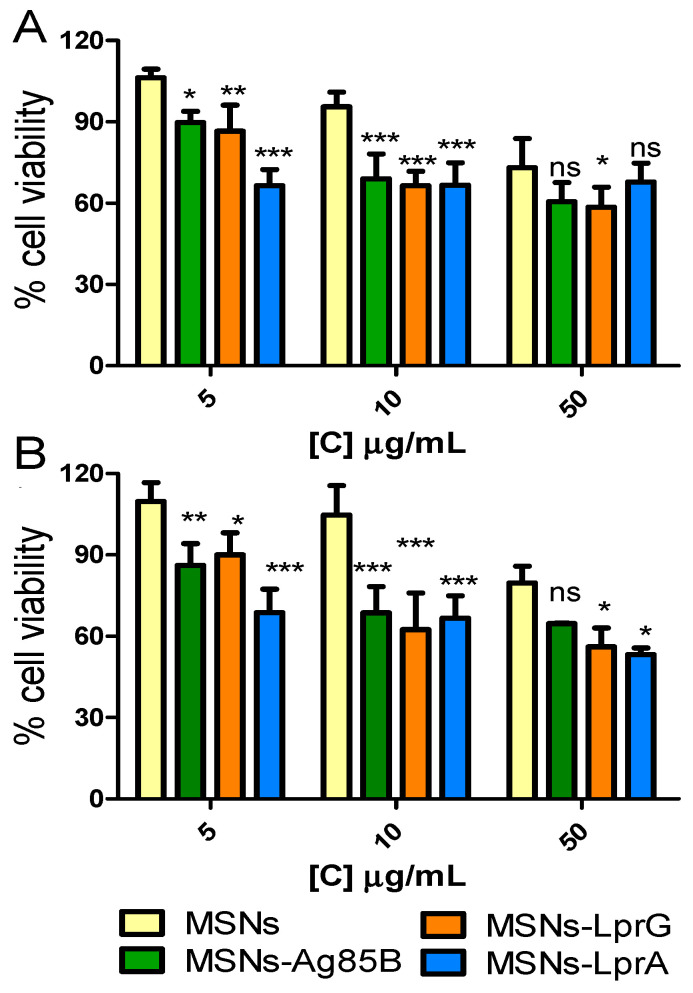
Cell viability of RAW 264.7 cells exposed to different concentrations (5, 10, 50 μg/mL) of MSNs, MSNs-Ag85B, MSNs-LprG, and MSNs-LprA for (**A**) 24 and (**B**) 48 h (*n* = 5). Data were evaluated by ANOVA followed by Bonferroni´s multiple-comparison test. *** *p* < 0.001, ** *p* < 0.01, and * *p* < 0.05 versus cells treated with MSNs; ns, not significant.

Cell viability was evaluated in the mouse macrophage cell line RAW 264.7 exposed to different concentrations (5, 10, and 50 μg/mL) of the MSNs, MSNs-Ag85B, MSNs-LprG, or MSNs-LprA nanosystems for 24 and 48 h. The results showed that in none of the cases viability was reduced by more than 50% (Figure 5). However, a decrease in cell viability was observed for the cells exposed to the materials with immunomodulatory proteins compared to cells exposed to the non-functionalized material (MSNs). The decrease in cell viability was dose-dependent, with no significant differences when comparing the two exposure times tested. The reduction in viability observed in macrophages can be explained by interference of the nanosystems with the signaling pathways involved in the activation of the immune response [28]. Based on these results, we selected the dose of 10 μg/mL for 24 h to evaluate the effects of the MSNs-Prot*_Mtb_* nanomaterials in the activation of the immune system as a dose that maintains biocompatibility in the in vitro study.

### 3.4. Evaluation of Immunostimulatory Capacity of MSNs-Prot_Mtb_ Nanosystems

It is widely documented that upon mycobacterial infection stimulated cells produce cytokines such as TNF, INF-γ, and IL-12 as part of an inflammatory Th1 response [47,48]. In a previous study, it was shown that stimulation of bone-marrow macrophages with native *Mtb* EVs did not triggered a typical Th1 response, including the induction of cytokines such as IL-10 which oppose Th1 priming [49]. Moreover, IL-10 has been shown to block activation of macrophages, which may benefit the intracellular fate of the bacteria and enhance their dissemination. In order to investigate the immunostimulatory capacity of the designed MSNs-Prot*_Mtb_* nanosystems, we measured the release of TNF and IL-10 by RAW 267.4 macrophages stimulated with 10 μg/mL of the nanosystems for 24 h. The study was performed under normal growth conditions, i.e., in the presence of complete culture medium, as well as in the absence of serum (i.e., starving conditions). These two different conditions were assayed to evaluate whether possible non-specific interactions or adsorption of serum proteins on the surface of the nanomaterials could affect the exposure of the *Mtb* proteins to the macrophage cells and; therefore, result in a variation in the secretion of the studied cytokines [50,51].

**Figure 6:**
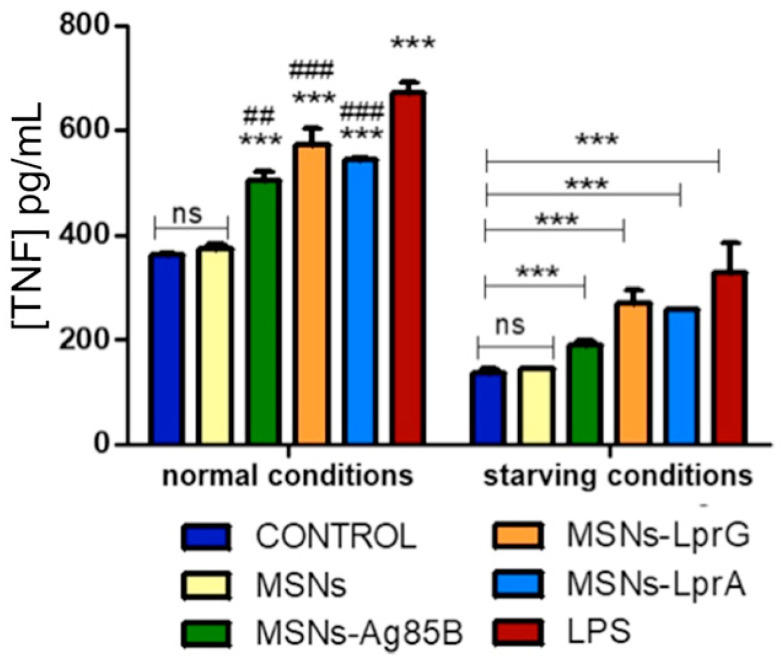
TNF levels after exposure of RAW 264.7 cells to 10 μg/mL of MSNs, MSNs-Ag85B, MSNs-LprG, or MSNs-LprA for 24 h (*n* = 3). Cells treated with lipopolysaccharide (LPS) (100 ng/mL) were used as a positive control. Data were evaluated by ANOVA followed by Bonferroni´s multiple-comparison test. *** *p* < 0.001 versus control; ^##^ *p* < 0.005 and ^###^ *p* < 0.001 versus cells treated with MSNs; ns, not significant.

**Figure 7:**
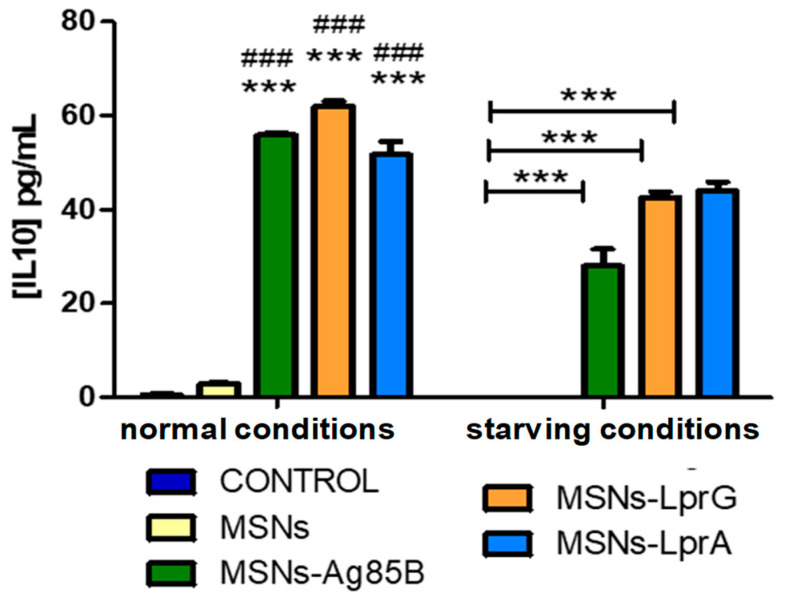
IL-10 levels after exposure of RAW 264.7 cells to 10 μg/mL of MSNs, MSNs-Ag85B, MSNs-LprG, and MSNs-LprA for 24 h (*n* = 3). IL-10 was not detected in cells treated with LPS. Data were evaluated by ANOVA followed by Bonferroni’s multiple-comparison test. *** *p* < 0.001 versus control; ^###^ *p* < 0.001 versus MSNs treated cells.

Exposure of the mouse macrophages to the MSNs-Prot*_Mtb_* nanosystems produced a significant secretion of TNF in the cultures compared to the control (Figure 6). The observed increase in the release of TNF was also significant with respect to cells exposed to the bare MSNs materials, thus confirming the additive effect of Ag85B, LprG and LprA proteins supported on the MSNs external surface. In addition, we found a comparable effect of the MSNs materials functionalized with the *Mtb* proteins in cells treated with LPS as a positive control for the release of TNF. Regarding the IL-10 assay, we found higher levels of this cytokine after stimulation with the materials bearing the *Mtb* proteins compared to the control, as well as to cells treated with the non-functionalized MSNs material (Figure 7). IL-10 was not detected in cells treated with LPS.

Based on these results, we can conclude that the developed nanosystems behave as the natural *Mtb* EVs in their capacity to induce both pro-inflammatory and anti-inflammatory cytokines.

## 4. Conclusions

The present work constitutes a proof of concept to evaluate the potential of mesoporous silica nanoparticles (MSNs) as a platform for the development of potential vaccines against tuberculosis. The approach has consisted in the functionalization of the external surface of the MSNs with immunomodulatory proteins (Ag85B, LprG and LprA) with the aim of developing nanosystems that mimic extracellular vesicles produced naturally by *Mtb*. The nanosystems have been extensively characterized, demonstrating the effectiveness of the covalent anchorage of the immunomodulatory proteins to the surface of the MSNs. All three nanosystems (MSNs-Ag85B, MSNs-LprG, and MSNs-LprA) showed a similar dose-dependent cytotoxicity in exposed macrophages that could be explained by the interference of the MSNs and the immunomodulatory proteins with the signaling pathways involved in the activation of the immune response. Exposure of the macrophages to the nanosystems produced a significant secretion of both the pro-inflammatory cytokine TNF and the anti-inflammatory cytokine IL-10 as compared to the control. The observed increase in the release of both cytokines was also significantly higher when comparing to cells exposed to bare MSNs, thus confirming the immunomodulatory effect of the anchored proteins. Due to the capacity of the designed nanosystems to induce pro-inflammatory and anti-inflammatory cytokines as the naturally produced *Mtb* EVs, on-going work is being focused on the feasibility of incorporating other vesicle components such as lipids or polysaccharides into the nanosystems. In this regard, the versatility of these nanosystems could be extended to the accommodation of antitubercular drugs within the mesopores of the MSNs so that they could provide dual function combining the immunomodulatory effect with a bactericidal action against *Mtb*.

## Figures and Tables

**Table 1:** Organic content from thermogravimetric analysis of MSNs and functionalized MSNs materials. Concentration of non-anchored protein from Bradford assay of protein functionalized MSNs.

Material	Org. Content (wt%)	[Protein*_Mtb_*] Nominal (μg/mL) ^2^	[Non-Anchored Protein*_Mtb_*] (μg/mL)	% Anchored Protein*_Mtb_*
MSNs	3.9	---	---	---
MSNs-COOH_ext_-CTAB	34.6 ^1^	---	---	---
MSNs-COOH_ext_	5.4 ^1^	---	---	---
MSNs-Ag85B	8.1 ^1^	91.9	0.64	99%
MSNs-LprG	8.7 ^1^	67.47	0.56	99%
MSNs-LprA	8.6 ^1^	71.96	3.89	95%

^1^ Organic content (wt%) was determined from the TGA weight losses, excluding the weight loss due to the desorption of water (up to 125 °C) and further corrected by the weight loss of the remaining alkoxysilanes after the sol-gel reaction (surfactant extracted unmodified MSNs). ^2^ The nominal concentration of each protein was calculated considering the amount of proteins utilized in the volume of reaction.

**Table 2:** **ζ**-potential values and hydrodynamic particle size in water medium of MSN materials.

Material	ζ-Potential (mV)	Hydrodynamic Size (nm)
MSNs	−19 ± 8	176 ± 13
MSNs-COOH_ext_	−28 ± 7	223 ± 29
MSNs-Ag85B	−24 ± 6	178 ± 18
MSNs-LprG	−27 ± 7	192 ± 18
MSNs-LprA	−25 ± 8	182 ± 20

**Table 3:** Chemical shifts and populations (%) of the silicon Q^n^ and T^n^ environments and peak area relations, (Q^2^ + Q^3^)/Q^4^ and Q^n^/T^n^ units, on the basis of the deconvolution of ^29^Si MAS NMR spectra of MSNs materials.

Material	δ, ppm (Peak Area, %)			Peak Area Ratio	δ (ppm)		Peak Area Ratio
	Q^2^	Q^3^	Q^4^	(Q^2^ + Q^3^)/Q^4^	T^2^	T^3^	Q/T
MSNs	−93.2 (6.2)	−102.3 (44.6)	−112.0 (49.1)	1.0	---	---	---
MSNs-COOH_ext_	−93.6 (3.2)	−102.7 (36.5)	−112.0 (60.2)	0.6	−57.8	−66.9	15.3
MSNs-LprG	−93.7 (2.7)	−102.6 (35.5)	−112.5 (61.8)	0.6	−57.3	−66.7	15.2

**Table 4:** Textural parameters of the MSN materials obtained by N_2_ adsorption measurements.

Material	S_BET_ (m^2^/g)	D_p_ (nm)	V_T_ (cm^3^/g)	V_P_ (cm^3^/g)
MSNs	1368.4	2.73	1.64	1.09
MSNs-CTAB	104.2	---	---	---
MSNs-COOH_ext_	557.2	2.03	0.391	0.274
MSNs-LprG	440.6	1.20	0.389	0.218

S_BET_ is the specific surface area obtained by using the BET equation, D_P_ is the pore diameter calculated by using the Barrett-Joyner-Halenda (BJH) method, V_T_ is the total pore volume obtained at P/P_0_ = 0.99, and V_P_ is the total pore volume obtained at P/P_0_ = 0.60.

## Supplementary Materials

The following are available online at https://www.mdpi.com/1999-4923/12/12/1218/s1, characterization techniques, synthesis and characterization of MSNs, Figure S1: MALDI-TOF mass spectra of (a) Ag85B, (b) LprG and, (c) LprA proteins, Figure S2: (a) SEM and (b) TEM images of the surfactant extracted MSNs material. (c) Low-angle powder X-ray diffraction pattern of the starting mesoporous material MSNs. (d) Table of XRD maxima indexed to the hk planes of p6mm 2D hexagonal mesoporous symmetry and their corresponding lattice spacing (d_hk_), Figure S3: (a) Zeta potential of MSNs and MSNs-Prot*_Mtb_* hybrid materials. Zeta potential analysis (four independent measurements) (b and c) and hydrodynamic size distribution obtained by dynamic light scattering (d and e) of MSNs-Ag85B and MSNs-LprG materials suspended in water media (MSNs-LprG as representative example of the lipoprotein functionalized material). Figure S4: ^13^C {^1^H} CP MAS NMR spectra of MSNs material. Peaks designated with # correspond to ethoxy groups due to incomplete hydrolysis and condensation.

## References

[1] World Health Organization (WHO) (2019). Global Tuberculosis Report.

[2] Kaufmann S.H. (2010). Future vaccination strategies against tuberculosis: Thinking outside the box. Immunity.

[3] Prados-Rosales R., Baena A., Martinez L.R., Luque-Garcia J.L., Kalscheuer R., Veeraraghavan U., Camara C., Nosanchuk J.D., Besra G.S., Chen B. (2011). Mycobacteria release active membrane vesicles that modulate immune responses in a TLR2-dependent manner in mice. J. Clin. Investig..

[4] Brown L., Wolf J., Prados-Rosales R., Casadevall A. (2015). Through the wall: Extracellular vesicles in Gram-positive bacteria, mycobacteria and fungi. Nat. Rev. Microbiol..

[5] Prados-Rosales R., Carreño L.J., Batist-Gonzalez A., Baena A., Venkataswamy M.M., Xu J., Yu X., Wallstrom G., Magee D.M., LaBaer J. (2014). Mycobacterial membrane vesicles administered systemically in mice induce a protective immune response to surface compartments of mycobacterium tuberculosis. mBio.

[6] Rodriguez G.M., Prados-Rosales R. (2016). Functions and importance of mycobacterial extracellular vesicles. Appl. Microbiol. Biotechnol..

[7] Mishra M., Kumar P., Rajawat J.S., Malik R., Sharma G., Modgil A. (2018). Nanotechnology: Revolutionizing the science of drug delivery. Curr. Pharm. Des..

[8] Wong I.Y., Bhatia S.N., Toner M. (2013). Nanotechnology: Emerging tools for biology and medicine. Genes Dev..

[9] Machuca A., Garcia-Calvo E., Anunciacao D.S., Luque-Garcia J.L. (2020). Rhodium nanoparticles as a novel photosensitizing agent in photodynamic therapy against cancer. Chem. Eur. J..

[10] Estevez H., Garcia-Lidon J.C., Luque-Garcia J.L., Camara C. (2014). Effects of chitosan-stabilized selenium nanoparticles on cell proliferation, apoptosis and cell cycle patter in HepG2 cells: Comparison with other selenospecies. Colloids Surf. B.

[11] Estevez H., Palacios A., Gil D., Anguita J., Vallet-Regi M., Gonzalez B., Prados-Rosales R., Luque-Garcia J.L. (2020). Antimycobacterial effect of selenium nanoparticles on mycobacterium tuberculosis. Frontiers Microbiol..

[12] Montalvo-Quirós S., Gomez-Graña S., Vallet-Regi M., Prados-Rosales R.C., Gonzalez B., Luque-Garcia J.L. (2021). Mesoporous silica nanoparticles containing silver as novel antimycobacterial agents against Mycobacterium tuberculosis. Colloids Surf. B.

[13] Costa-Gouveia J., Aínsa J.A., Brodin P., Lucía A. (2017). How can nanoparticles contribute to antituberculosis therapy?. Drug Discov. Today.

[14] Ballester M., Nembrini C., Dhar N., De Titta A., De Piano C., Pasquier M., Simeoni E., Van Der Vlies A.J., McKinney J.D., Hubbell J.A. (2011). Nanoparticle conjugation and pulmonary delivery enhance the protective efficacy of Ag85B and CpG against tuberculosis. Vaccine.

[15] Villegas M.R., Baeza A., Vallet-Regí M. (2018). Nanotechnological strategies for protein delivery. Molecules.

[16] Vallet-Regí M., Ramila A., Del Real R.P., Pérez-Pariente J. (2001). A new property of MCM-41: Drug delivery system. Chem. Mater..

[17] Castillo R., Lozano D., González B., Manzano M., Izquierdo I., Vallet-Regí M. (2019). Advances in mesoporous silica nanoparticles for targeted stimuli-responsive drug delivery: An update. Expert Opin. Drug. Deliv..

[18] Manzano M., Vallet-Regí M. (2020). Mesoporous silica nanoparticles for drug delivery. Adv. Funct. Mater..

[19] Moreno V.M., Álvarez E., Izquierdo-Barba I., Baeza A., Serrano-López J., Vallet-Regí M. (2020). Bacteria as nanoparticles carrier for enhancing penetration in a tumoral matrix model. Adv. Mater. Interfaces.

[20] Lu J., Liong M., Li Z., Zink J.I., Tamanoi F. (2010). Biocompatibility, biodistribution, and drug-delivery efficiency of mesoporous silica nanoparticles for cancer therapy in animals. Small.

[21] Xu C., Lei C., Yu C. (2019). Mesoporous silica nanoparticles for protein protection and delivery. Front. Chem..

[22] Montalvo-Quirós S., Aragoneses-Cazorla G., García-Alcalde L., Vallet-Regí M., González B., Luque-García J.L. (2019). Cancer cell targeting and therapeutic delivery of silver nanoparticles by mesoporous silica nanocarriers: Insights into the action mechanisms using quantitative proteomics. Nanoscale.

[23] Villegas M.R., Baeza A., Vallet Regí M. (2015). Hybrid collagenase nanocapsules for enhanced nanocarrier penetration in tumoral tissues. ACS Appl. Mater. Interfaces.

[24] Villegas M.R., Baeza A., Noureddine A., Durfee P., Butler K., Agola J., Brinker J.C., Vallet-Regí M. (2018). Multifunctional protocells for enhanced penetration in 3D extracellular tumoral matrices. Chem. Mater..

[25] Clemens D.L., Lee B.-Y., Xue M., Thomas C.R., Meng H., Ferris D., Nel A.E., Zink J.I., Horwitz M.A. (2012). Targeted intracellular delivery of antituberculosis drugs to mycobacterium tuberculosis-infected macrophages via functionalized mesoporous silica nanoparticles. Antimicrob. Agents Chemother..

[26] Hwang A.A., Lee B.-Y., Clemens D.L., Dillon B.J., Zink J.I., Horwitz M.A. (2015). pH-Responsive Isoniazid-Loaded Nanoparticles Markedly Improve Tuberculosis Treatment in Mice. Small.

[27] Li X., Xue M., Raabe O.G., Aaron H.L., Eisen E.A., Evans J.E., Hayes F.A., Inaga S., Tagmount A., Takeuchi M. (2015). Aerosol droplet delivery of mesoporous silica nanoparticles: A strategy for respiratory-based therapeutics. Nanomedicine.

[28] Mody K.T., Popat A., Mahony D., Cavallaro A.S., Yu C., Mitter N. (2013). Mesoporous silica nanoparticles as antigen carriers and adjuvants for vaccine delivery. Nanoscale.

[29] Carvalho L.V., Ruiz R., Scaramuzzi K., Marengo E.B., Matos J.R., Tambourgi D.V., Fantini M.C., Sant’Anna O.A. (2010). Immunological parameters related to the adjuvant effect of the ordered mesoporous silica SBA-15. Vaccine.

[30] Liu T., Liu H., Fu C., Li L., Chen D., Zhang Y., Tang F. (2013). Silica nanorattle with enhanced protein loading: A potential vaccine adjuvant. J. Colloid Interface Sci..

[31] Skrastina D., Petrovskis I., Lieknina I., Bogans J., Renhofa R., Ose V., Dishlers A., Dekhtyar Y., Pumpens P. (2014). Silica nanoparticles as the adjuvant for the immunisation of mice using hepatitis B core virus-like particles. PLoS ONE.

[32] Lu Y., Yang Y., Gu Z., Zhang J., Song H., Xiang G., Yu C. (2018). Glutathione-depletion mesoporous organosilica nanoparticles as a self-adjuvant and co-delivery platform for enhanced cancer immunotherapy. Biomaterials.

[33] Du G., Woythe L., Van Der Maaden K., Leone M., Romeijn S., Kros A., Kersten G., Jiskoot W., Bouwstra J.A. (2018). Coated and Hollow Microneedle-Mediated Intradermal Immunization in Mice with Diphtheria Toxoid Loaded Mesoporous Silica Nanoparticles. Pharm. Res..

[34] Oliveira D.C.D.P., De Barros A.L.B., Belardi R.M., De Goes A.M., Souza B.K.D.O., Soares D.C.F. (2016). Mesoporous silica nanoparticles as a potential vaccine adjuvant against Schistosoma mansoni. J. Drug Deliv. Sci. Technol..

[35] Aguilar-López B.A., Correa F., Moreno-Altamirano M.M.B., Espitia C., Hernandez-Longoria R., Oliva-Ramirez J., Padierna-Olivos J., Sancgez-Garcia F.J. (2019). LprG and PE_PGRS33 Mycobacterium tuberculosis virulence factors induce differential mitochondrial dynamics in macropahges. Scan J. Immunol..

[36] Martínez A., Fuentes-Paniagua E., Baeza A., Sánchez-Nieves J., Cicuéndez M., Gómez R., de la Mata F.J., González B., Vallet-Regí M. (2015). Mesoporous silica nanoparticles decorated with carbosilane dendrons as new non-viral oligonucleotide delivery carriers. Chem. Eur. J..

[37] Lee J., Kim S.H., Choi D.S., Lee J.S., Kim D.K., Go G., Park S.M., Kim S.H., Shin J.H., Chang C.L. (2015). Proteomic analysis of extracellular vesicles derived from Mycobacterium tuberculosis. Proteomics.

[38] Lim M., Stein A. (1999). Comparative studies of grafting and direct syntheses of inorganic-organic hybrid mesoporous materials. Chem. Mater..

[39] de Juan F., Ruiz-Hitzky E. (2000). Selective functionalization of mesoporous silica. Adv. Mater..

[40] IEP_Ag85B_ Calculated from the Protein Sequence NCBI GenBank (Ag85B, A5U3Q3.1) and the Expasy Program. https://web.expasy.org/protparam/.

[41] IEP_LprG_ Calculated from the Protein Sequence NCBI GenBank (LprG, ANZ82088.1) and the Expasy Program. https://web.expasy.org/protparam/.

[42] IEP_LprA_ Calculated from the Protein Sequence NCBI GenBank (LprA, ANZ81937.1) and the Expasy Program. https://web.expasy.org/protparam/.

[43] Luo Z., Hu Y., Xin R., Zhang B., Li J., Ding X., Hou Y., Yang L., Cai K. (2014). Surface functionalized mesoporous silica nanoparticles with natural proteins for reduced immunotoxicity. J. Biomed. Mater. Res. Part A.

[44] Nieto A., Colilla M., Balas F., Vallet-Regí M. (2010). Surface electrochemistry of mesoporous silicas as a key factor in the design of tailored delivery devices. Langmuir.

[45] Rosenholm J.M., Sahlgren C., Lindén M. (2010). Towards multifunctional, targeted drug delivery systems using mesoporous silica nanoparticles—Opportunities & challenges. Nanoscale.

[46] González B., Colilla M., Díez J., Pedraza D., Guembe M., Izquierdo-Barba I., Vallet-Regí M. (2018). Mesoporous silica nanoparticles decorated with polycationic dendrimers for infection treatment. Acta Biomater..

[47] Chensue S.W., Warmington K., Ruth J., Lincoln P., Kuo M.C., Kunkel S.L. (1994). Cytokine responses during mycobacterial and schistosomal antigen-induced pulmonary granuloma formation. Production of Th1 and Th2 cytokines and relative contribution of tumor necrosis factor. Am. J. Pathol..

[48] Chensue S.W., Warmington K.S., Ruth J.H., Lincoln P., Kunkel S.L. (1995). Cytokine function during mycobacterial and schistosomal antigen-induced pulmonary granuloma formation. Local and regional participation of IFN-gamma, IL-10 and TNF. J. Immunol..

[49] Gordon S. (2003). Alternative activation of macrophages. Nat. Rev. Immunol..

[50] Lesniak A., Fenaroli F., Monopoli M.P., Åberg C., Dawson K.A., Salvati A. (2012). Effects of the Presence or Absence of a Protein Corona on Silica Nanoparticle Uptake and Impact on Cells. ACS Nano.

[51] Salvati A., Pitek A.S., Monopoli M.P., Prapainop K., Baldelli Bombelli F., Hristov D.R., Kelly P.M., Åberg C., Mahon E., Dawson K.A. (2013). Transferrin-functionalized nanoparticles lose their targeting capabilities when a biomolecule corona adsorbs on the surface. Nat. Nanotechnol..

