# Supplementary material for: Mesoporous Silica Nanoparticles as a Potential Platform for Vaccine Development against Tuberculosis
Source: Pharmaceutics. 2020 Dec 16;12(12):1218. doi: 10.3390/pharmaceutics12121218 (PMC7767215; doi:10.3390/pharmaceutics12121218)
Supplement: Supplementary file 1 [file pharmaceutics-12-01218-s001.pdf]

pharmaceutics

Supplementary Materials: Mesoporous Silica Nanoparticles as a Potential Platform for Vaccine Development against Tuberculosis

Sandra Montalvo-Quirós, María Vallet-Regí, Ainhoa Palacios, Juan Anguita, Rafael C. Prados-Rosales, Blanca González and Jose L. Luque-Garcia

Characterization techniques

Matrix-assisted laser desorption/ionization time-of-flight mass spectrometry (MALDI-TOF MS, Kyoto, Japan) was used to characterize MTB proteins before conjugation onto MSNs. The isolated proteins were dissolved in MES buffer (50 mM) and synaptic acid was used as matrix. Prior to the analysis of proteins, MES buffer was measured using the same method to confirm possible interferences. Protein characterization was evaluated in a range of 10,000-100,000 m/z. Mass spectra were acquired in an Ultraflex MALDI-TOF mass spectrometer (Bruker, Billerica, MA, USA).

Thermogravimetric analysis (TGA) and differential thermal analysis (DTA) were performed in a Perkin Elmer Pyris Diamond TG/DTA analyser (Perkin Elmer, Waltham, CA, USA) by placing approximately 5 mg of sample in an aluminium crucible and applying 5 °C/min heating ramps from room temperature to 600 °C under a flow rate of 100 mL/min of air. Chemical microanalyses were performed with a Perkin Elmer 2400 CHN and a LECO CHNS-932 thermoanalyzers.

Solid state magic angle spinning nuclear magnetic resonance (MAS NMR) and cross polarization magic angle spinning nuclear magnetic resonance (CP MAS NMR) spectra were obtained on a Bruker Avance AV-400WB spectrometer equipped with a solid state probe using a 4 mm zirconia rotor. Typical measurement conditions were as follows: ^{13}C NMR experiments were conducted with proton decoupling, the spectrometer frequency was set to 100.62 MHz (^{13}C) and 400.13 MHz (^{1}H) and MAS rotation rate was maintained at 6 kHz. The NMR spectra consisted of 15,000 to 17,500 acquisitions with cross-polarization contact times of 2 ms, 2.5 μs pulse wide and a 5 s recycle delay. Chemical shift values were referenced at 176.1 ppm to carbonyl carbon of glycine. ^{29}Si NMR quantitative spectra were obtained at 79.49 MHz with MAS rotation rate of 12 kHz, a pulse wide of 4.5 μs, a recycle delay of 5 s, and the number of scans was 17,000 to 20,000. 29Si CP MAS NMR experiments were done setting the transmitter frequency to 79.49 MHz (^{29}Si) and 400.13 MHz (^{1}H), samples were spun to 12 kHz and a 5 s recycle delay, a 4.5 μs pulse wide and a cross-polarization contact time of 3.5 ms were employed. All chemical shift values reported were externally referenced at 0 ppm to 3-(trimethylsilyl)-1-propanesulfonic acid sodium salt (DSS sodium salt).

The powder X-ray diffraction (XRD) measurements were performed in a Philips X′Pert diffractometer with Bragg-Brentano geometry operating with Cu Kα radiation (wavelength 1.5406 Å) at 40 kV and 20 mA (Philips Electronics NV, Eindhoven, Netherlands). Low-angle XRD patterns were collected in the 2θ range between 0.6° and 8° with a step size of 0.02° and contact time of 5 s per step.

The textural properties of the materials were determined by N2 adsorption porosimetry by using a Micromeritics ASAP 2020 (Micromeritics Co., Norcross, GA, USA). To perform the N2 measurements, ca. 30 mg of each sample was previously degassed under vacuum for 24 h at 40 °C. The surface area (SBET) was determined using the Brunauer-Emmett-Teller (BET) method and the pore volume (VP) was estimated from the amount of N2 adsorbed at a relative pressure of 0.98. The pore size distribution between 0.5 and 40 nm was calculated from the desorption branch of the isotherm by means of the Barrett-Joyner-Halenda (BJH) method and the average mesopore size (D_P) was determined from the maximum of the pore size distribution curve.

Electrophoretic mobility measurements of the materials suspended in water were used to calculate the zeta-potential (ζ) values of the nanoparticles. Measurements were performed in a Zetasizer Nano ZS (Malvern Instruments Ltd., Malvern, United Kingdom) equipped with a 633 nm

"red" laser. For this purpose, 1 mg of nanoparticles was added to 10 mL of water followed by vortex and ultrasound to get a homogeneous suspension. Measurements were recorded by placing ca. 1 mL of suspension in DTS1070 disposable folded capillary cells (Malvern Instruments). The hydrodynamic size of nanoparticles was measured by dynamic light scattering (DLS) with the same Malvern instrument. Values presented are mean ±SD from triplicate measurements.

Surface morphology was analysed by scanning electron microscopy (SEM) on a JEOL JSM 6335F microscope (Tokyo, Japan). For this purpose, samples were mounted onto a copper stud, dried at 70 °C for 48 h under vacuum and coated with a film of Au previous to observation.

Transmission Electron Microscopy (TEM) was carried out with a JEOL JEM 2100 instrument operated at 200 kV (JEOL Ltd., Tokyo, Japan). Sample preparation was performed by dispersing ca. 1 mg of sample in 1 mL of 1-butanol followed by sonication in a low power bath sonicator (Selecta, Spain) for 5 min and then depositing one drop of the suspension onto carbon-coated copper grids.

Synthesis and characterization of MSNs

MSNs. Fluorescent MSNs were prepared following a modified Stöber method in which the fluorescent dye was covalently linked to the silica network [1,2,3]. APTS (5 μL, 0.023 mmol) was added over a stirred FITC (2 mg, 0.005 mmol) solution in EtOH (0.25 mL), under N_2 atmosphere. The reaction was stirred for 2 h in the dark at RT and then added to a solution containing TEOS (5 mL, 0.023 mol) and EtOH (1 mL). The resulting solution was subsequently placed on a srynge dispenser to be transferred to the next reaction. The structure directing agent CTAB (1 g, 2.74 mmol) was dissolved in 480 mL of water and 3.75 mL of NaOH 2 M and the solution heated to 80 °C under gently stirring. At that point, the solution containing TEOS and the silane-modified fluorescein was added dropwise at a constant rate of 0.43 mL/min under vigorous stirring. The reaction was vigorously stirred for 1 h at 80 °C in the dark and then the suspension was cooled to room temperature, centrifuged and the particles washed with water, EtOH and finally dried.

The SEM analysis revealed that the obtained MSNs had uniform spherical shape with an average particle diameter of ca. 150 nm, and TEM images showed a highly ordered mesopore network of the MSNs (Figure S2A and S2B, respectively). Low-angle XRD measurement showed a well-resolved characteristic pattern indexed to a p6mm symmetry of 2D hexagonal MCM-41 materials with a unit cell parameter of 4.46 nm ($a_0 = 2/3 \sqrt{3}\ d_{10}$) (Figure S2C). The well-ordered mesoporous structure was also confirmed by N_2 adsorption porosimetry, exhibiting type IV isotherms which were analysed by BET and BJH methods to determine a surface area of 1368.4 m^2/g and average pore diameter of 2.73 nm, respectively (see Figure 4 and Table 4 in manuscript).

MSNs-$COOH_{ext}$. The external surface functionalization of MSNs with carboxylic acids was performed onto the pore surfactant containing material (34.6% wt.). Approximately a quarter of the specific surface area of the free-surfactant material (1368.4 m2/g) was considered to be functionalized [1]. First, 1 g of CTAB-containing MSNs (0.654 g MSNs) was dehydrated at 80 °C under vacuum for 3 h in the dark. Subsequently, TESPSA (0.2032 g, 10% exc.) was dissolved in 15 mL of dry toluene and added to a vigorously stirred suspension of the CTABcontaining MSNs dispersed in dry toluene (60 mL), under N_2 atmosphere. The reaction mixture was heated to 110 °C overnight in the dark, afterwards it was centrifuged and the modified MSNs were exhaustively washed with toluene and acetone and finally dried. The surfactant extraction was carried out by heating a well dispersed suspension of obtained solid in EtOH (360 mL), water (40 mL) and HCl (10 mL) overnight at 60 °C and then the solid was washed with water and EtOH. This process was repeated for 2 h and the solid dried under vacuum. The experimental value of –COOH groups in this material is 6.74×10^{-4} mol/g MSN-$COOH_{ext}$, calculated from the organic content (5.4%) due to the functionalization, as determined by termogravimetry.

To provide anchoring points for the proteins, the external surface of the MSNs was functionalized with carboxylic acid groups in a first step, using a post-synthesis method. The condensation of the succinic anhydride alkoxysilane derivative TESPSA with the silanol groups of the outer silica surface, under water free conditions [4], was performed using the assynthesized MSN material containing the organic template filling the pores. With this methodology, a favoured

functionalization of the outer surface of MSNs is foreseeable [5]. The surfactant was removed afterwards using acidified ethanol as extracting solution, affording in the same stage the anhydride ring opening. The required amount of alkoxysilane derivative was calculated to achieve a maximum coverage of the external nanoparticle surface (a 100% nominal degree of functionalization). For this, approximately a quarter of the specific surface area was estimated to correspond to the external surface and a molar ratio of three Si-OH groups with one $R\text{-}Si(OEt)_3$ molecule was the stoichiometry used. In addition, it was presumed that the average surface concentration of Si-OH in silica materials is 4.9 OH/nm^2 [6].

References

1. World A. Martínez, E. Fuentes-Paniagua, A. Baeza, J. Sánchez-Nieves, M. Cicuéndez, R. Gómez, F. J. de la Mata, B. González and M. Vallet-Regí. *Chem. Eur. J.* **2015**, *21*, 15651-15666. Mesoporous Silica Nanoparticles Decorated with Carbosilane Dendrons as New Non-viral Oligonucleotide Delivery Carriers.
2. V. Y.-S. Lin, C.-P. Tsai, H.-Y. Huang, C.-T. Kuo, Y. Hung, D.-M. Huang, Y.-C. Chen and C.-Y. Mou. *Chem. Mater.* **2005**, *17*, 4570-4573. Well-ordered Mesoporous Silica Nanoparticles as Cell.
3. M. Grün, I. Lauer and K. K. Unger. *Adv. Mater.* **1997**, *9*, 254-257. The Synthesis of Micrometerand Submicrometer-Size Spheres of Ordered Mesoporous Oxide MCM-41.
4. M. Lim and A. Stein, *Chem. Mater.*, 1999, **11**, 3285-3295. Comparative Studies of Grafting and Direct Syntheses of Inorganic-Organic Hybrid Mesoporous Materials.
5. F. de Juan and E. Ruiz-Hitzky, *Adv. Mater.*, 2000, **12**, 430-432. Selective Functionalization of Mesoporous Silica.
6. L. T. Zhuravlev, *Colloids Surfaces A Physicochem. Eng. Asp.*, 2000, **173**, 1-38. The Surface Chemistry of Amorphous Silica. Zhuravlev Model.

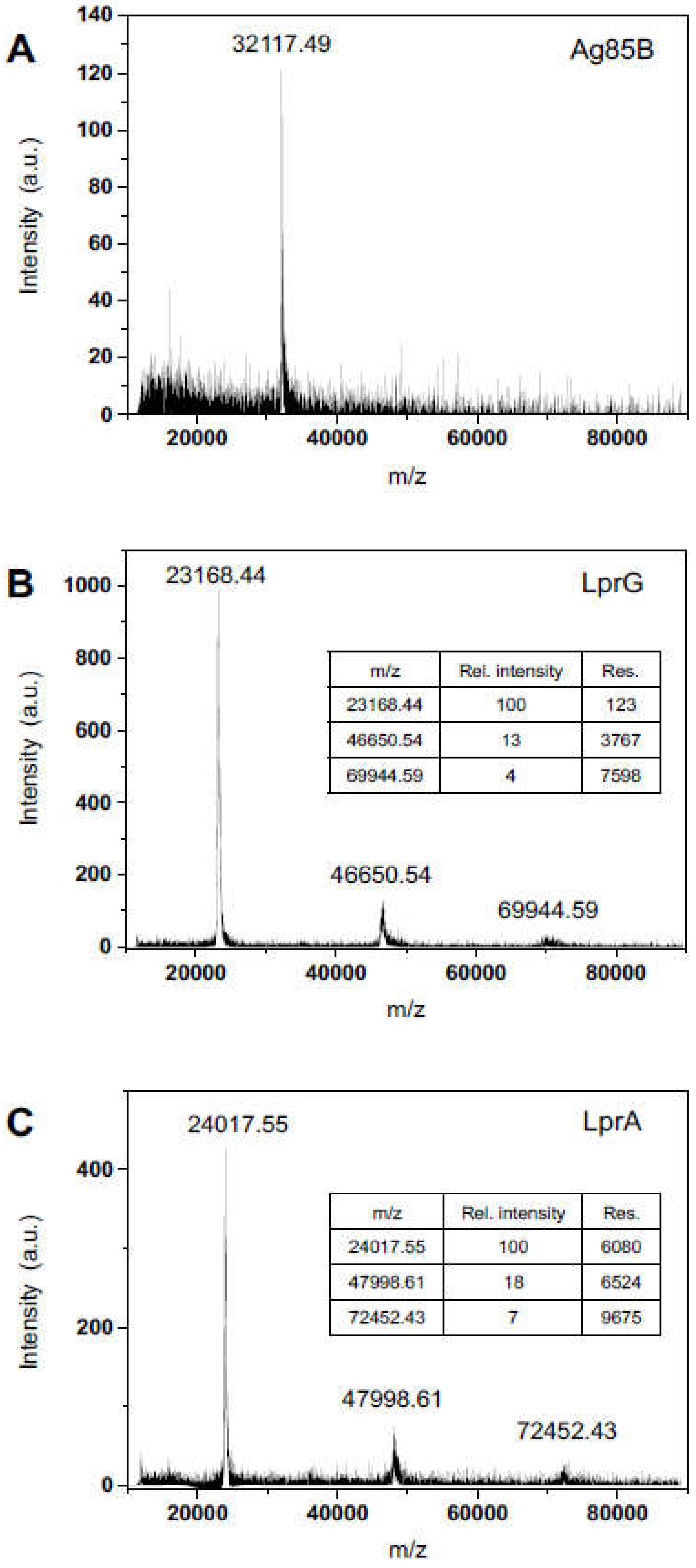

Figure S1. MALDI-TOF mass spectra of **A**) Ag85B, **B**) LprG and **C**) LprA proteins.

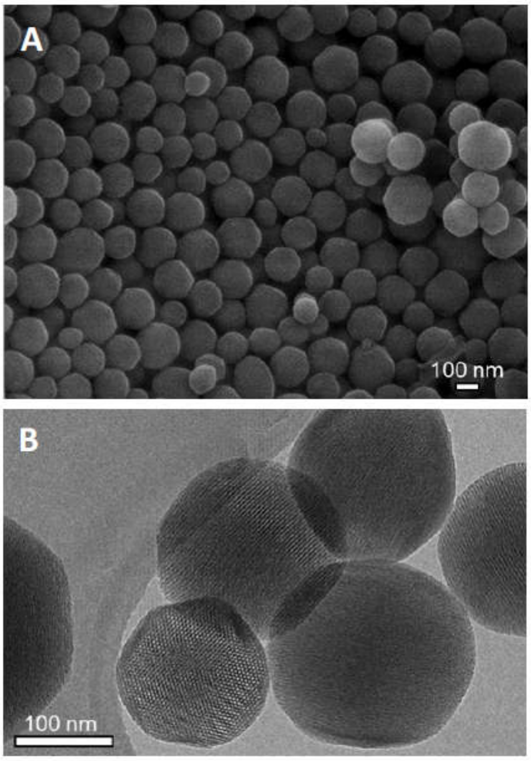

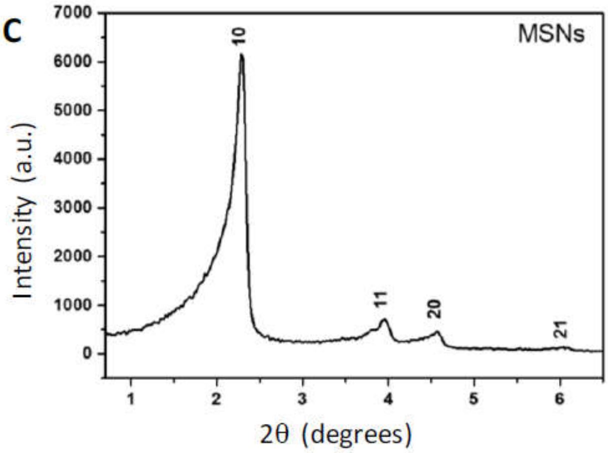

D

(*hk*)	2θ	d_{hk} (nm)
10	2.28	3.864
11	3.95	2.235
20	4.56	1.932
21	6.03	1.462

Figure S2. A) SEM and **B**) TEM images of the surfactant extracted MSNs material. **C**) Low angle powder X-ray diffraction pattern of the starting mesoporous material MSNs. **D**) Table of XRD maxima indexed to the hk planes of *p6mm* 2D hexagonal mesoporous symmetry and their corresponding lattice spacing (dhk).

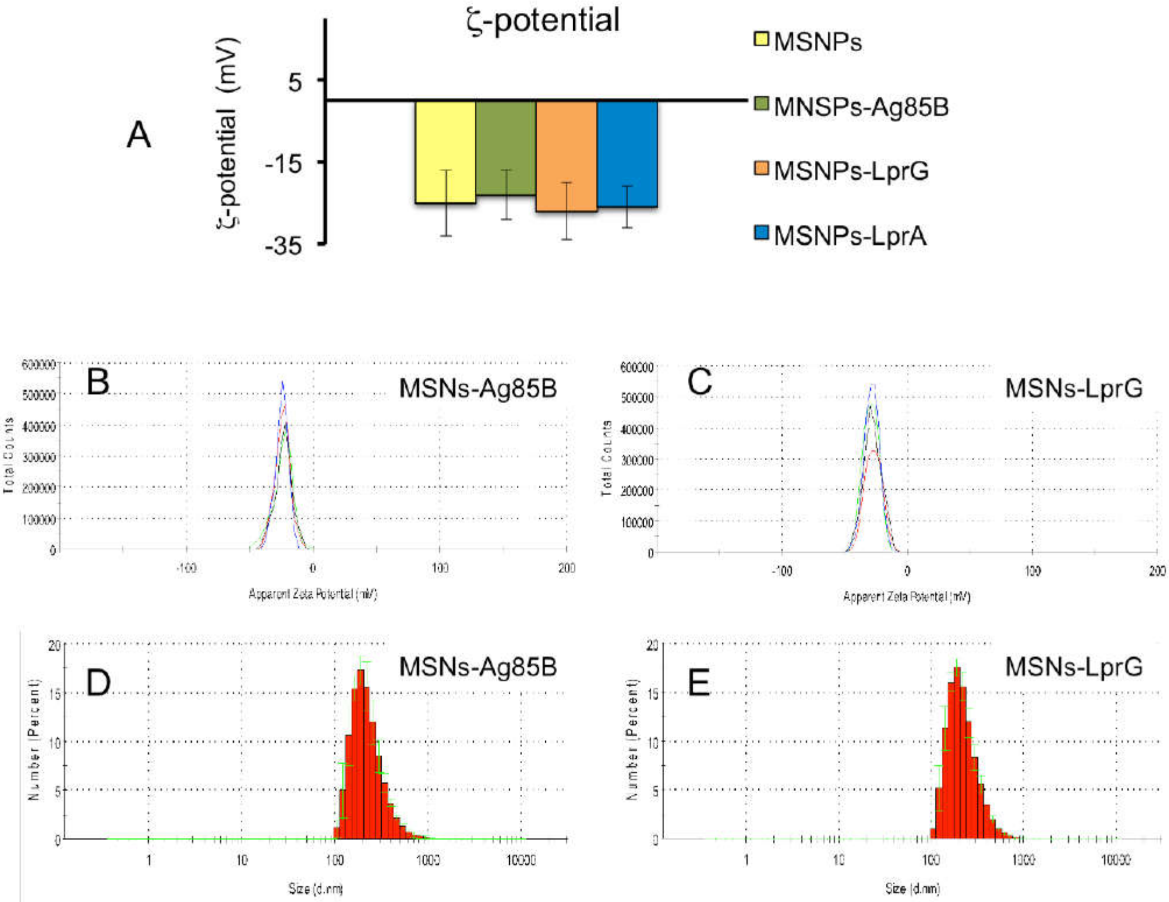

Figure S3. A) Zeta potential of MSNs and MSNs-ProtMtb hybrid materials. Zeta potential.analysis (four independent measurements) (**B** and **C**) and hydrodynamic size distribution obtained by dynamic light scattering (**D** and **E**) of MSNs-Ag85B and MSNs-LprG materials suspended in water media (MSNs-LprG as representative example of the lipoprotein functionalized material).

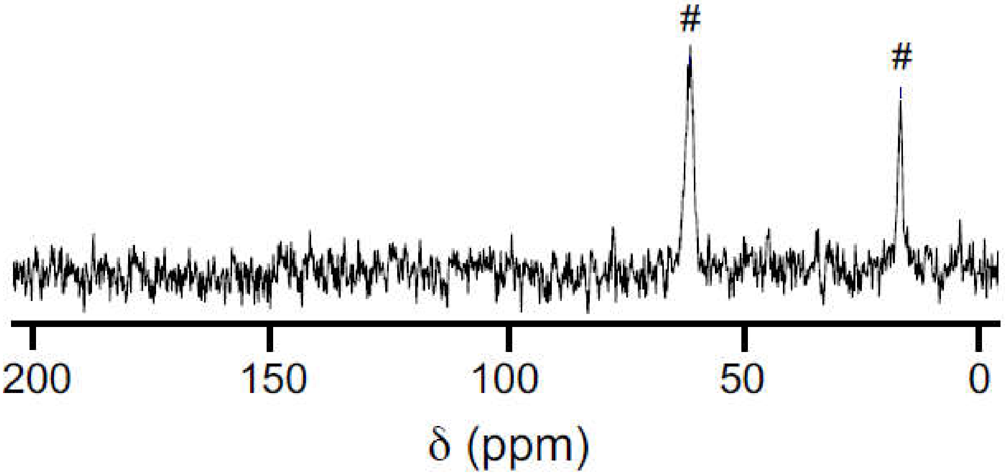

Figure S4. ^{13}C {^{1}H} CP MAS NMR spectra of MSNs material. Peaks designated with # correspond to ethoxy groups due to incomplete hydrolysis and condensation.

© 2020 by the authors. Submitted for possible open access publication under the terms and conditions of the Creative Commons Attribution (CC BY) license (http://creativecommons.org/licenses/by/4.0/).
